# An Integrative Model of Effortful Control

**DOI:** 10.3389/fnsys.2019.00079

**Published:** 2019-12-20

**Authors:** Nathalie André, Michel Audiffren, Roy F. Baumeister

**Affiliations:** ^1^Research Centre on Cognition and Learning, UMR CNRS 7295, University of Poitiers, Poitiers, France; ^2^School of Psychology, University of Queensland, Brisbane, QLD, Australia

**Keywords:** cost-benefit, fatigue, mental effort, network connectivity, resources, salience network, self-control, theta rhythm

## Abstract

This article presents an integrative model of effortful control, a resource-limited top-down control mechanism involved in mental tasks and physical exercises. Based on recent findings in the fields of neuroscience, social psychology and cognitive psychology, this model posits the intrinsic costs related to a weakening of the connectivity of neural networks underpinning effortful control as the main cause of mental fatigue in long and high-demanding tasks. In this framework, effort reflects three different inter-related aspects of the same construct. First, effort is a mechanism comprising a limited number of interconnected processing units that integrate information regarding the task constraints and subject’s state. Second, effort is the main output of this mechanism, namely, the effort signal that modulates neuronal activity in brain regions involved in the current task to select pertinent information. Third, effort is a feeling that emerges in awareness during effortful tasks and reflects the costs associated with goal-directed behavior. Finally, the model opens new avenues for research investigating effortful control at the behavioral and neurophysiological levels.

## Introduction

Although effort is used to explain a large variety of phenomena, it is very difficult to find a clear definition of this concept (Massin, 2017). Since the beginning of experimental psychology, the effort has been associated with voluntary attention and will. For instance, William James conceived the terms attention and effort as two names for the same psychic fact depending on the brain processes and emanating from the Self (James, 1918). Subsequently, the concept of effort has remained a topic of interest in the field of psychology until the 21th century and is still a subject of studies and debates (e.g., Westbrook et al., 2019). The main purpose of this article is to provide a theoretical framework that more precisely defines the concept of effort and specifies the neurophysiological bases and neurobiological mechanisms explaining its possible weakening with acute fatigue. This framework integrates knowledge from cognitive-energetic models, psychosocial models and advances in the field of neuroscience.

Overall, in this framework, effort reflects three different inter-related aspects of the same construct. First, effort is a mechanism comprising a limited number of interconnected processing units that integrate information regarding the task constraints, rewards and subject’s state. We conceive these processing units as the cortical minicolumns belonging to several cortical areas taking part in a large functional neuronal network called the Salience Network.

Second, effort is the main output of this mechanism, namely, the effort signal that modulates neuronal activity in brain regions involved in the current task to select pertinent information. We assume that the mechanism of effort exerts its control over other task-related brain regions through a slow (4–8 Hz) rhythmical effort signal generated by the pyramidal neurons of the Salience Network.

Third, effort is a feeling that emerges in awareness during effortful tasks and reflects the costs associated with goal-directed behavior. We support the view that the simultaneous activation of interconnected pyramidal neurons in layers 2–3 of cortical minicolumns belonging to the Salience network mainly contributes to a global workspace supporting effort awareness.

Finally, we propose that the capacity of the Salience Network to generate the effort signal can be weakened by short-term synaptic mechanisms, leading to the feeling of mental fatigue and to a performance drop. “Definitions of Key Concepts” section describes more extensively this integrative model of effortful control. “Neurophysiological Arguments for an Integrative Model of Effortful Control” section presents evidence from neuroscience research supporting this model. “Challenging the Integrative Model of Effortful Control” section proposes some experimental designs to validate hypotheses inferred from this new model of effort.

### Resource vs. Cost-Benefit Models

Effortful tasks include maintaining concentration on complex problem solving, sustaining attention on infrequent cues, repressing urges, running at an uncomfortable intensity, and many other activities. These tasks are frequently performed in sport situations (e.g., endurance races), social situations (e.g., self-control tasks) and work situations (e.g., vigilance tasks). These tasks are often perceived as costly, difficult and sometimes uncomfortable, unpleasant or aversive (Kurzban, 2016; Hsu et al., 2018; Inzlicht et al., 2018).

The mechanism explaining the disengagement of effort during a task is still under debate. Two main theoretical approaches have been used to explain the decrement in performance observed in experiments using the sequential-task paradigm (Lee et al., 2016) or during vigilance tasks over time. The first approach refers to cost-benefit models, which explain performance declines, dropouts, and withdrawals in terms of a shift toward a more valuable, rewarded or pleasant behavior (Kurzban et al., 2013; Inzlicht et al., 2014; Shenhav et al., 2017). According to this first perspective, fatigue can be conceived as a cost. The nature of the costs leading an individual to stop or decrease the intensity of commitment is extensively discussed. Several types of costs have been identified as follows: energetic costs (Boksem and Tops, 2008), intrinsic costs (Shenhav et al., 2017) and opportunity costs (Kurzban et al., 2013). The second approach refers to resource models, which primarily attribute decreases in performance to a decline in available resources (Baumeister et al., 2007; Warm et al., 2008). According to this second perspective, fatigue is conceived as a state of depleted resources.

Cost-benefit models disagree regarding the prevalent costs, whereas resources models fail to clearly define the depleted resources. Some researchers have proposed that blood glucose and astrocyte glycogen are possible candidates as depletable resources (Baumeister et al., 2007; Gailliot et al., 2007; Christie and Schrater, 2015; Baumeister and Vohs, 2016), but these hypotheses have been challenged and criticized (Kurzban et al., 2013; Inzlicht and Berkman, 2015; Shenhav et al., 2017; although see Ampel et al., 2018). Alternative theories based on cost-benefit analyses and motivational change have similarly received severe criticisms, including the gradual accumulation of contradictory findings (e.g., Baumeister and Vohs, 2016).

The integrative model of effortful control attempts to reconcile both approaches. On the one hand, we agree with cost-benefit models that the decision-making process regarding the deployment of effort is based on a cost-benefit analysis. However, we disagree regarding the nature of the costs in the computation. We assume that the weight of the different possible costs changes according to situational demands. For instance, in a marathon race, energetic costs are very high, whereas, in a vigilance task lasting 60 min, these costs are considerably lower compared to computational costs depending on stimulus saliency, target probability, and their psycho-physical properties. Furthermore, the opportunity cost of performing some behavior is very high when individuals have the free choice to select among several possible valuable alternatives. For instance, the opportunity cost of solving a math problem is higher in the presence of a smartphone, because this situation provides an opportunity to play with the smartphone (Kurzban et al., 2013). However, the opportunity cost might be low or close to zero under some circumstances, such as when only one response could avoid immediate death.

Finally, we agree with resource models that in long tasks overloading executive functions there is a progressive decrease in the capacity to exert effortful control. However, we propose a new perspective regarding the nature of the resource that decreases overtime during the task. Instead of a fuel that can be depleted through prolonged use, the resource may be the connectivity of a neural network that can be weakened through intensive use. In addition, we assume that this decrease in capacity constitutes a cost associated with mental fatigue.

### Mental and Physical Effort

The use of the term “effort” is often associated with physical exertion as well as mental exertion (Massin, 2017). The univocal nature of effort is questioned as follows: should effort be considered a single construct that can be applied in the same way to cognitive tasks and physical exercises or should we differentiate these two types of effort, i.e., one type that is only relevant to cognitive activities named “mental effort” or “cognitive effort” and another type that is only relevant to physical activities named “physical effort”.

We assume that physical effort is physical exertion that requires mental effort to maintain intensity for the duration of the task. For instance, some physical activities require very minimal effort (e.g., automatic skills practiced at the most comfortable pace, such as jogging at any easy speed) and some physical activities require much effort (e.g., new skills or skills practiced at a high intensity). However, there are some important differences between mental and physical fatigue. In particular, physical (muscular) fatigue can reach the point at which the muscle is simply unable to function, whereas that has not been shown with mental fatigue.

It is also important to distinguish between effort and energy expenditure expressed in calories. Kahneman (1973) distinguished these two concepts as follows: “the momentary effort that a task demands must be distinguished from the total amount of work that is required to complete that task. The momentary effort exerted in running the 60-yard dash is greater than the effort exerted in walking two miles at a comfortable pace, although the total expenditure is surely greater in the second task” (p. 25). Consequently, the effort is viewed here as a single construct involved in the regulation of the intensity of behavior regardless of the nature of the activities required to reach the intended goal, i.e., mental, physical or both. Presenting arguments for and against this univocal nature of the effort is beyond the scope of this article.

In summary, this article addresses the following questions: What exactly is effort? What brain regions support effortful control? Can these mechanisms be weakened by acute uses of overloaded effortful control?

## Definitions of Key Concepts

This section delineates our integrative model of “effort”, which is a critical intervening variable involved in mental tasks and physical exertion. The first aim of this model is to propose a framework explaining the decrements in performance and acute mental fatigue effects observed following an exhausting self-control task (Hagger et al., 2010) or throughout long vigilance tasks (See et al., 1995; Warm et al., 2008). The second aim of this model is to more clearly define the different types of resources solicited in effortful self-control tasks. All hypotheses formulated in this framework are testable at the behavioral or neurophysiological level.

**Figure 1:**
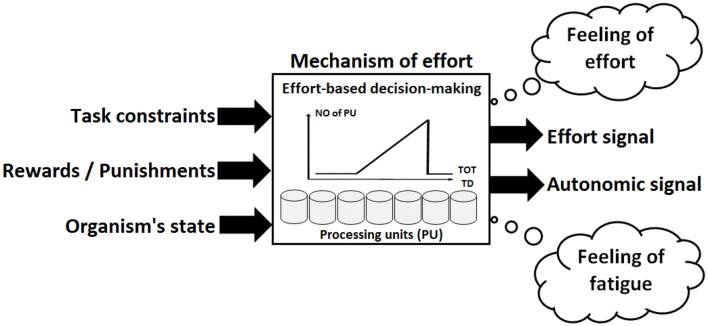
Schematic representation of the integrative model of effortful control. The construct of effort involves the following three aspects: (1) the mechanism of effort, which is a neural network including a finite number of processing units (PU); (2) an effort signal generated by these processing units; and (3) a feeling of effort raising consciousness once the signal of effort is generated. The mechanism of effort fulfills the main function of making decisions regarding the intensity and the direction of the engagement in effort in ongoing or future tasks. Decisions are determined by the integration of miscellaneous input signals related to the task constraints (e.g., number of alternatives), immediate and delayed rewards (e.g., financial gain, social approval, or satisfaction of biological urge) or punishments (e.g., financial loss, social disapproval, or painful experience), and the current state of the organism (e.g., level of arousal or level of blood glucose). The mechanism of effort produces two main outputs: the effort signal and the autonomic signal. The strength of the effort signal depends on the number of processing units recruited to achieve the goal of the task. As the task difficulty (TD) and/or time on task (TOT) increases, the number of processing units recruited increases proportionally. The effort signal exerts control over task-related brain regions, helping them select relevant task features and inhibit irrelevant ones. The autonomic signal is sent to the sympathetic system to mobilize energy. The feelings of effort would correspond to the awareness of the costs required to achieve the goal of the task. The feeling of fatigue would correspond to the awareness of the high intrinsic costs that could prevent the organism from coping with a future threat.

In our model, which is described in Figure 1, “effort” reflects three inter-related aspects of the same neuropsychological construct. First, “effort” is a resource-limited top-down control mechanism, i.e., a neural network comprising interconnected and distributed neuronal assemblies fulfilling the functions of decision-making, coping with stressful situations, and controlling other brain regions involved in cognitive tasks and physical exercises. We use the term “mechanism of effort” to refer to this aspect of “effort”.

Second, “effort” is a control signal, i.e., an electrophysiological signal generated by the mechanism of effort that is sent to brain regions involved in the achievement of the planned goal. We use the term “effort signal” to denote this aspect of the effort. The effort signal is the main output of the mechanism of effort. The effort signal is sent to specialized brain regions to inhibit irrelevant information and select pertinent task-related information to perform the task.

Third, “effort” is a feeling, i.e., a perception computed from the different costs associated with goal-directed behavior. We use the term “feeling of effort” to denote this aspect of the effort. The following sections more precisely describe each of the three facets of effort.

Finally, the processing units that compute and generate the effort signal within the mechanism of effort are the heart of the integrative model of effortful control. We conceive the brain as an assembly of highly interconnected processing units functioning in parallel to contribute to the continuous processing of information to produce adapted behaviors. These processing units have a specific function that depends on their localization in the brain and the type of information they receive. For instance, the processing units localized in the primary visual cortex are specialized in the processing of visual information. Similarly, we assume that a pool of processing units belonging to the mechanism of effort is specialized in computing and generating the effort signal according to cost and benefit signals. Throughout the manuscript, we use the term “effort-dedicated processing units” to designate this pool of specialized processing units. The connectivity between several processing units reflects the capacity of these processing units to communicate, exchange information and work in synchronization with other processing units.

### Mechanism of Effort as a Limited-Capacity System

We assume that the mechanism of effort is systematically activated once an individual engages in a mental task or a physical exertion. We imagine a continuum from effortless tasks, i.e., highly automatized tasks, to very effortful tasks, such as cognitive tasks dominantly tapping executive functions, self-control or voluntary attention and very intense or painful exercises. The amount of effort cannot be deployed ad infinitum in an extreme effortful task, i.e., the mechanism of effort is a resource- or capacity-limited mechanism.

In order to illustrate how much effort is dedicated to a task, let us take the example of watching a movie with two different instructions. In the first condition, the participants would be instructed to watch an emotionally neutral movie showing animals living in their natural environment and to answer some simple questions on the content of the movie at the end of the task. In this condition, many areas in the brain would be activated to have a good perception of the visual scenes and to store pertinent information in long-term memory in order to answer the questions at the end of the movie. This task is relatively easy and typically does not require a large amount of effort. Consequently, our model predicts that brain areas involved in the mechanism of effort would be weakly activated and few effort-dedicated processing units would be recruited. In the second condition, the participants would watch a very sad movie with emotionally evocative scenes—after being instructed to suppress any feeling and behavioral manifestation of sadness. This second task requires emotional inhibition and much more effortful control than the first. In this case, our model predicts that the brain regions involved in the mechanism of effort would be much more activated than in the first task and that more effort-dedicated processing units would be recruited. By contrast, brain areas involved in visual processing and storage of information in long-term memory will be activated quite similarly in both tasks.

We assume that the capacity of the mechanism of effort is limited due to two main reasons: (1) the number of processing units composing the mechanism is finite; and (2) the connectivity of the neuronal network underpinning the mechanism of effort, and consequently its capacity to generate the effort signal, can be weakened when solicited strongly and/or at length.

The first idea related to the notion of limited capacity assumes that the mechanism of effort includes a finite number of processing units devoted to the main function of generating a control signal. According to this first meaning, the word “capacity” refers to the maximum number of processing units available for participating in the mechanism of effort. Moreover, since the number of processing units is finite, only a limited set of processing units belonging to the mechanism of effort can be allocated to a task or distributed among several tasks. Thus, processing units can only work on one task feature at a time.

Therefore, we assume that when one task is completed, these units become available for another task. This assumption requires that processing units of the mechanism of effort are task non-specific. In that respect, the assumption seemingly clashes with considerable evidence from the animal literature. Indeed, many studies showed that neurons belong to specialized categories with response properties making them suitable for particular classes of computations, such as neurons in sensory areas that code for task parameters, for instance, the direction of motion in visual areas (e.g., Britten et al., 1996). However, other studies suggest that neurons can also be category-free (e.g., Raposo et al., 2014). In that case, parameters of a task would be distributed randomly across neurons (Ganguli and Sompolinsky, 2012). This category-free selectivity has been observed in the orbitofrontal cortex and the dorsal anterior cingulate cortex (ACC), two brain structures involved in effort-based decision making (Blanchard et al., 2018). This category-free property confers flexibility, allowing the brain to use the same neuronal network to participate in multiple behaviors simply by using the same neurons in different ways, depending on the needs of the individual (Raposo et al., 2014).

According to the radial unit model (Rakic, 1988, 2009; see “Modular Organization of the Dorsal ACC and Anterior Insula Cortices” section), which explains how the neocortex develops, the number of processing units (i.e., cortical minicolumns) devoted to the mechanism of effort in a given individual is determined during the ontogenesis of the neocortex through interactions between genetic and environmental factors. However, this initial potential of the processing units may decrease due to strokes, concussions or neurodegenerative diseases that may occur throughout the lifespan. Consequently, each individual possesses a finite number of processing units composing his/her mechanism of effort at a specific age according to his/her personal history.

Furthermore, when the mechanism of effort has to cope with a single task, all processing units are available to achieve the unique goal of the task, but the task can usually be completed with only a subset of processing units. In the case of multiple tasks, the mechanism of effort has to distribute its total number of processing units among the different tasks according to a prioritization strategy. In accordance with cost-benefit approaches, we assume that the prioritization rules rely on the costs and benefits associated with each task that must be performed.

The second idea related to the notion of limited capacity assumes that the capacity of the mechanism of effort to generate the effort signal can be negatively impacted by a strong and/or long utilization of the effort-dedicated processing units. According to this second meaning, the word “capacity” refers to the capability of effort-dedicated processing units to generate the effort signal. Based on this perspective, the longer and stronger the generation of the effort signal, the higher the likelihood that this capacity is weakened overtime during the task. These fatigue-like effects indicate that even when a task is completed, there is a delay before the connectivity within the neuronal network underpinning the mechanism of effort retrieves its optimal state. An optimal state of connectivity means that all effort-dedicated processing units can generate the effort signal with high efficiency.

A direct consequence of a weakening of the effort signal is that if an individual wishes to maintain the level of performance despite the decrement in the efficiency of the processing units recruited to generate the effort signal, he/she must recruit additional processing units (i.e., invest more effort) to compensate for that loss of efficiency.

We propose that the transitory decrement in the capacity to generate the effort signal relies on a change in the electrophysiological properties of the prefrontal pyramidal neurons involved in the solicited processing units rather than a depletion of an intrinsic biological fuel, such as brain glucose. These molecular mechanisms are detailed in “Neurobiological Mechanisms Underlying Acute Mental Fatigue” section and result in a decreased capacity of prefrontal pyramidal neurons belonging to effort-dedicated processing units to generate the effort signal, i.e., an alteration of their firing rate.

Brain glucose can play different roles in the effort-based decision-making process. First, resupplying carbohydrates can decrease the perception of metabolic costs (e.g., sports drinks including carbohydrates during long runs such as marathons and ultra-marathons). Second, glucose can participate in the weakening of the processing units devoted to effort exertion because high consumption of glucose leads to the production of adenosine, which is a metabolite that modulates the electrophysiological properties of neurons. This new conception of the limited-capacity of exerting effortful control is an alternative to the glucose fuel model (e.g., Baumeister et al., 2007).

Our model keeps close to the main ideas of resource models (Baumeister et al., 1998; Warm et al., 2008), which assumes a decrease in the capacity to exert effortful control in the case of long and high loading of executive functions. By contrast, we assume that this detrimental mechanism relies on the production of metabolites (e.g., adenosine) that impair the capacity to exert effortful control. The short-term changes occurring within the processing units after their long and/or overloaded solicitation are extensively described in “Neurobiological Mechanisms Underlying Acute Mental Fatigue” section.

### Functions of the Mechanism of Effort

The mechanism of effort has two main functions. First, the mechanism of effort makes decisions regarding acting or not acting, maintaining or stopping an activity, and choosing one activity among several activities. As previously mentioned, the output of the decision-making process is a control signal enabling the selection of the appropriate behavior. To generate this signal, the decision-making process needs to integrate numerous miscellaneous signals providing information regarding the costs and benefits of ongoing and future possible activities.

Costs can be defined as factors that have detrimental consequences on an organism at a physical or psychological level while attempting to achieve an intended goal. Costs change according to the task demands and constraints. When demands and constraints increase, costs increase. Costs also depend on the amount of extrinsic or intrinsic resources expended to achieve a goal (e.g., the amount of money to buy a car or number of calories expended to run a marathon). Based on this perspective, costs are relative to the number of resources owned by an individual and the value he/she attributes to this resource. For instance, the cost of running a marathon is likely perceived higher by a novice runner than an elite athlete. Similarly, the cost of a fancy meal could be perceived very differently by different people (e.g., a homeless person vs. a millionaire) and in different circumstances (e.g., a bank account that is close to zero vs. bank account on one’s payday).

Benefits refer to factors that have positive consequences on an organism if the goal is achieved. The immediate or delayed satisfaction of a need, approach of a positive experience, or avoidance of a negative experience due to the achievement of a goal can be viewed as examples of benefits. The computation and prioritization of costs and benefits require information regarding the current state of the organism (e.g., level of blood glucose, level of arousal, or level of pain in a part of the body) and the detection or anticipation of any serious deviation from homeostasis and well-being representing a need of or threat to the organism. Consequently, we hypothesize that the neural network underpinning the mechanism of effort should integrate various multi-modal cost and benefit signals originating from numerous miscellaneous brain regions.

The other main function of the mechanism of effort allows an organism to cope with current or anticipated stressful situations, i.e., situations that carry a potential cost. Based on this perspective, the mechanism of effort maintains task performance under disturbance from stressors. Consequently, we hypothesize that the activation of the neuronal network underpinning the mechanism of effort should increase with task difficulty (TD). More precisely, in accordance with Kukla (1972), Brehm and Self (1989) and Brouwer et al. (2014), we assume that the number of processing units recruited by the mechanism of effort to perform the task is a linear function of the perceived TD, up to a limit. This upper limit is determined by whether the values of the needs and objectives justify the amount of effort required.

Coping with a situation requires the allocation of the resources necessary to achieve the intended goal. Consequently, we assume that the mechanism of effort also plays a pivotal role in allostasis and the generation of allostatic responses (Sterling, 2004; Juster et al., 2010), i.e., maintaining physiological stability by matching the parameters of the internal milieu with environmental demands. Therefore, the mechanism of effort must exert control over Cannon’s well-known “fight-or-flight” system (hypothalamo-sympathoadrenal system) and Selye’s “stress” system (hypothalamopituitary-adrenal system) to manage the amount of energy dedicated to the task.

Finally, in accordance with Sanders’s (1983, 1997) and Hockey’s (1993, 1997, 2005, 2011) cognitive-energetic models of effort, we assume that the mechanism of effort compensates for the suboptimal states of an organism by recruiting more processing units to maintain the output necessary to achieve high-priority task goals. Suboptimal states of an organism include low levels of arousal (i.e., reduced noradrenergic activity), few perceived benefits (i.e., reduced dopaminergic activity) and a decrease in the capacity to exert effortful control with the current recruited units (i.e., reduced connectivity in the neuronal network underpinning the mechanism of effort).

### The Effort Signal

The previous section asserted that the mechanism of effort includes a finite number of processing units. The function of each effort-dedicated processing unit is to generate a control signal that helps targeted brain regions involved in carrying out the current task to keep the focus on relevant task features (see “The Effort Signal as a Product of the Synchronized and Rhythmic Firing of Pyramidal Neurons” section for more details).

As previously mentioned, the effort signal results from the integration of a large variety of signals conveying the costs and benefits associated with the likelihood of success and failure in achieving the goal of the task. Each processing unit belonging to the mechanism of effort achieves this integration. Once generated, the effort signal is sent to brain regions involved in carrying out the task and helps these regions focus on relevant task features and avoid interference by irrelevant information by modulating their neuronal activity.

### The Feeling of Effort

Humans experience feelings of effort while performing effortful tasks. Once the task is completed, the feeling of effort subsides. However, the costs associated with the ongoing task certainly leave a trace in long-term memory, allowing the individual to rate how much effort he/she deployed in the completed task. Here, we assume that the feeling of effort is related to physical exercises and mental tasks. We do not reduce the feeling of effort to the perception of the intensity of the motor output. Therefore, we restrict the use of the term “perception of effort,” which is also known as “perceived exertion” or “sense of effort,” quite exclusively to the feeling of effort during physical exercises.

In the present model, the feeling of effort is related to the awareness of how costly it is to achieve the goal of the task regardless of the nature of the task and the importance of the motor component of that task. The feeling of effort can be conceived as a perception based, at least in part, on the activity of processing units devoted to the deployment of effort in an ongoing activity. We assume that exerting effortful control directly contributes to the feeling of effort. Based on this perspective, the generation of the effort signal by the mechanism of effort might coincide with the occurrence of the feeling of effort in awareness. This view is consistent with the results obtained by Bijleveld (2018) in a working memory task. Thus, we assume that the larger the number of processing units involved in the generation of effortful control and the higher the activity required of these processing units to generate the control signal, the stronger the feeling of effort.

### The Feeling of Fatigue

Our model assumes that the decrement in effort capacity following a long and intense engagement in effort relies mainly on a change in the electrophysiological properties of the prefrontal pyramidal neurons involved in the processing units recruited to generate the effort signal. This weakening of the effort capacity can be viewed as a decrease in available resources, i.e., the capacity of the processing units to generate the effort signal, an increase in internal costs, and a decrease in effort capacity necessitating the recruitment of more processing units to maintain performance. In this context, we assume that the feeling of fatigue is related to the awareness of how much the capacity of the organism to perform the task is weakened at a given moment and how much it could be detrimental for the organism to continue with the same intensity of engagement.

Consistent with Benoit et al. (2019), we conceive the feeling of fatigue as a warning signal urging the participant to stop the task or decrease the intensity of engagement in anticipation of future adverse consequences. The feeling of fatigue is essential for maintaining a person’s physical integrity (Ament and Verkerke, 2009). We assume that the following signals contribute to the emergence of the awareness of the feeling of fatigue overtime during a task: (1) inflation of energetic and/or computational costs; (2) negative feedback regarding task performance; and (3) alarming physiological states.

The costs change according to the task demands and constraints. As the demands increase, the costs increase. The energetic costs become inflated when individuals expend much energy (i.e., calories) to perform a strenuous physical exercise. For instance, climbing Mount Everest taxes more resources than walking to the nearest pub. Furthermore, computational costs (i.e., the load exerted on effort-dedicated processing units) become inflated when individuals overload executive functions for a long time. For example, performing a simultaneous translation for 30-min taxes more resources than dreaming of the upcoming holidays.

## Neurophysiological Arguments for an Integrative Model of Effortful Control

In this section, we describe the neurobiological substrate of the mechanism of effort, the structural elements that generate and maintain the effort signal over time, the main characteristics of this control signal, and the neurophysiological mechanisms that underpin the phenomenon of acute fatigue.

### Searching for the Neural Network Underlying the Mechanism of Effort

The main goal of this section is to identify the neural network that could generate and send the effort signal to other brain structures to achieve an intended goal. Consistent with Dehaene et al. (1998) and Dehaene and Changeux (2011), we assume that the brain processes underlying effortful tasks require a unique global workspace comprising distributed and heavily interconnected neurons in different brain regions. The neural network that constitutes this global workspace needs to satisfy the five following conditions of appropriateness to be identified as the mechanism of effort.

First, the neural activity within this network should increase as the TD required to perform the task increases; the higher the TD, the higher the electrochemical activity of this network until the upper limit of effort disengagement is reached. TD is certainly the most well-studied task constraint associated with effort deployment (Kukla, 1972; Kahneman, 1973; Brehm and Self, 1989; Brouwer et al., 2014). We do not assume here that neurons involved in the mechanism of effort are the only neural cells coding for TD in the brain. Several studies showed that TD signals are represented broadly in the brain (e.g., Churchland et al., 2008). We rather assume that because effortful control increases with TD, the activity of neurons involved in the mechanism of effort must increase with TD.

Second, this neural network should integrate numerous and miscellaneous cost-benefit signals. On the one hand, this network must receive signals regarding endogenous homeostasis (e.g., core temperature, level of blood glucose), allowing this network to anticipate or react to any serious deviation representing a threat (i.e., a cost) to the organism, such as a high level of fatigue. On the other hand, this network must also receive reward signals (or expected rewards) certainly through a dopaminergic pathway.

Third, the control signal generated by this neural network should be closely related to the output of the sympathetic system, which is considered the gold standard for effort measurement; the higher the engagement in effort, the higher the demand in energy, and the higher the output of the sympathetic system.

Fourth, the sending of the control signal generated by this neural network should precede the activation of other networks involved in task performance.

Fifth, any lesion or dysfunction in this network should lead to a lack of mental energy and willpower, resulting in the failure in making decisions and detecting conflicts.

Neuroimaging studies analyzing resting-state functional connectivity have suggested the existence of at least three large-scale brain networks related to different aspects of high-level cognitive functions and self-regulation (Greicius et al., 2003; Fox et al., 2005, 2006; Seeley et al., 2007). These networks or systems include the Default-Mode Network (DMN), the Central Executive Network (CEN) and the Salience Network.

The DMN is a task-negative system that is deactivated during cognitively demanding tasks (Shulman et al., 1997; Binder et al., 1999; Mazoyer et al., 2001; McKiernan et al., 2003), while the other two networks are task-positive systems that are activated during a large variety of tasks (Seeley et al., 2007; Dosenbach et al., 2008). The DMN mainly includes the ventromedial prefrontal cortex, the posterior cingulate cortex, the precuneus, the retrosplenial cortex, the lateral parietal lobes and the medial temporal lobes (Gusnard and Raichle, 2001). The DMN plays a key role in self-related processes, introspection, self-awareness, metacognition, prospective self-projection, and autobiographic memory recall (Gusnard et al., 2001; Buckner and Carroll, 2007; Spreng and Grady, 2010; Salomon et al., 2014; Davey et al., 2016; Lou et al., 2017).

The CEN (Seeley et al., 2007), which shares many commonalities with the Fronto-Parietal Control Network (Dosenbach et al., 2008), contributes to executive control particularly by maintaining and updating information in working memory, sustained attention, response selection, and response suppression. The CEN is mainly anchored in the dorsolateral prefrontal cortex, the ventrolateral prefrontal cortex, the dorsomedial prefrontal cortex, and the lateral parietal cortex (Seeley et al., 2007; Dosenbach et al., 2008).

Finally, the Salience Network is involved in identifying the most homeostatically relevant signals among a myriad of internal and extrapersonal stimuli to make decisions (Seeley et al., 2007; Uddin, 2014), manage errors and conflicts (Menon et al., 2001; Kerns et al., 2004; Ridderinkhof et al., 2004), and ensure autonomic control (Thayer et al., 2009; Critchley et al., 2013). This system mainly consists of the orbital frontoinsular cortex, the dorsal ACC, the anterior insula, and the superior temporal gyrus (Seeley et al., 2007). The Salience Network has some similarities to the Cingulo-Opercular Network proposed by Dosenbach et al. (2008).

Among these three large-scale networks, the Salience Network satisfies all the criteria of appropriateness previously noted as a plausible substrate underlying the mechanism of effort. In the following section, we present evidence supporting each appropriateness criterion.

### Five Lines of Evidence

The first line of evidence concerns the positive relationship between TD and the activation of the Salience Network. According to this extensive literature, it is expected that the higher the difficulty of the task, the higher the activation of the brain structures involved in the Salience Network. A review conducted by Paus et al. (1998) examined 107 blood flow activation studies carried out with positron emission tomography (PET). In total, 1132 healthy volunteers were scanned in the 107 experiments reviewed. A significant increase in cerebral blood flow (CBF) was frequently observed in the dorsal ACC when the task was more difficult. More recently, six studies using functional magnetic resonance imaging (fMRI) reported a clear relationship between TD and the activation of the dorsal ACC and/or anterior insula (Laurienti et al., 2003; Engström et al., 2013; Shenhav et al., 2014; Wisniewski et al., 2015; Lamichhane et al., 2016; Shenhav et al., 2016). In summary, we can find clear arguments supporting the existence of a close relationship between the activity of two important nodes in the Salience Network (the dorsal ACC and anterior insula) and TD.

The second line of evidence concerns the diversity of the cost-benefit signals received by the Salience Network. On the one hand, if one of the main functions of the Salience Network is to cope with stressful situations, its core constituting elements should receive signals regarding the current state of the organism (e.g., interoceptive feelings or pain level). These signals inform the organism about potentials costs (e.g., the risk of discomfort or injury). The role of this defensive system may be to avoid any potentially dangerous events that threaten the body’s integrity or homeostasis or conserve energy for an upcoming and more rewarding activity. Based on this perspective, the anterior insula, which is an important node in the Salience Network, is well known to integrate interoceptive and pain signals (Craig, 2009). For instance, ascending lamina I activity related to pain is integrated into the anterior insula and ACC (Craig, 2003). In addition, fMRI and PET studies involving humans indicate that concomitant activity in the anterior insula and ACC occurs during the experience of virtually all emotions (Craig, 2002; Phan et al., 2002). In particular, subjective ratings of pleasant and unpleasant feelings in the body are directly correlated with lateralized representations of homeostatic afferent activity in the right anterior insula and ventrolateral prefrontal cortex. Therefore, the right anterior insula and the ACC represent a lateralized neurobiological substrate of the subjective awareness of arousing emotions (Craig, 2002). Thus, it is plausible to assume that the feelings of effort and fatigue, which are two cost-related feelings, emerge from the neuronal activity in the right anterior insula and the ACC.

**Figure 2:**
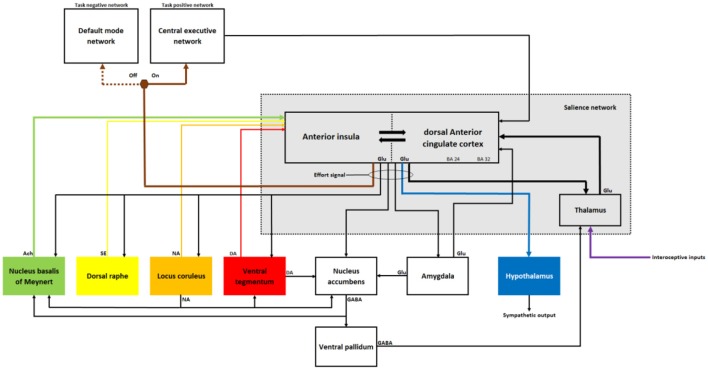
Schematic illustration of the key structures and pathways of the Salience Network that underlie the mechanism of effort. The illustration depicts and emphasizes certain direct pathways between structures or networks based on anatomical evidence of the main functions of the model presented in this article. The two main nodes of the salience network are the anterior insula and the dorsal anterior cingulate cortex(ACC). These two areas of the neocortex participate in the limbic system and are reciprocally interconnected. The Salience Network is represented by a gray rectangle. Once an individual is engaged in the realization of an effortful cognitive task or physical exercise, the Salience Network activates the Central Executive Network (CEN) and deactivates the default mode network through the effort signal sent by the pyramidal neurons in layer 5 of the cortical columns in the anterior insula (brown arrow). During the task, the Salience Network controls the activity of the CEN through the effort signal, which oscillates according to a theta rhythm. The pyramidal neurons located in layers 2–3 of the cortical columns in the anterior insula and dorsal ACC compute the effort signal. Once computed, the effort signal is propagated into neighboring columns through lateral connections (not represented) and pyramidal neurons in layer 5. These neurons integrate three essential inputs for the computation of the effort signal: (1) intrinsic inhibitory inputs from GABAergic interneurons in the same cortical columns (not represented); (2) excitatory inputs from glutamatergic neurons in the thalamus; and (3) inhibitory inputs from cholinergic neurons in the nucleus basalis of Meynert. Excitatory inputs from the thalamus ensure bursts of spikes. Inhibitory inputs from GABAergic interneurons and cholinergic neurons ensure periods of inhibition (green arrow). Interoceptive inputs informing the salience network about the state of the organism reach layers 2–3 of the anterior insula and dorsal ACC *via* the thalamus (purple arrow). The activity of the pyramidal neurons in the anterior insula and dorsal ACC is also modulated by several inputs that greatly influence the capacity of the Salience network to generate the effort signal. These inputs include the following: (1) dopaminergic inputs from neurons in the ventral tegmentum (red arrow); (2) noradrenergic inputs from neurons in the locus coeruleus (orange arrow); and (3) serotoninergic inputs from neurons in the raphe nucleus (yellow arrow). A downregulation or upregulation of these neurotransmitter systems could weaken the capacity of the Salience Network to generate and maintain the effort signal. The effort signal is also transmitted to the hypothalamus, which represents the main output hub of the sympathetic system (blue arrow). Finally, the reward system, including the ventral tegmentum, the nucleus accumbens, the ventral pallidum, the amygdala, and the thalamus modulates the effort signal according to the rewarding value of the task goal.

On the other hand, the computation carried out within the Salience Network should be modulated by information regarding rewards and incentives (i.e., benefits). Many existing studies investigating effort have shown that rewards generally increase the amount of effort an individual or animal invests to achieve the task goal (for a review on the monetary incentives on effort, see Bonner and Sprinkle, 2002). According to self-stimulation, pharmacological, physiological and behavioral studies, the ventral tegmentum, the nucleus accumbens, and the ACC clearly participate in a cortico-basal ganglia circuit that is the heart of the reward system (Hikosaka et al., 2008; Haber and Knutson, 2010). The ventral tegmentum is one of the two main sources of brain dopamine (Grimm et al., 2004). The target sites of the ventral tegmental area include several regions in the limbic system, including the nucleus accumbens, the amygdala and the ACC, and widespread regions in the neocortex with a higher projection density to the prefrontal cortex (Mehta and Riedel, 2006). The projections of the ventral tegmental area to the limbic system (i.e., the mesolimbic system; see Figure 2) have been associated with “wanting” behaviors (Berridge and Robinson, 1998; Robinson et al., 2016), which are characterized as a disposition to overcome costs to obtain an incentive or a greater reward (Kurniawan et al., 2011). Other authors have suggested that the tonic mode of spike firing in mesolimbic dopaminergic neurons leads to an invigoration of motivated behavior when faced with increasing demands of effort (Niv et al., 2007). More specifically, numerous neuroscientists have established that reward signals from the mesolimbic dopamine system modulate the activity of the ACC to optimize the response selection process (for reviews, see Botvinick et al., 2004; Nieuwenhuis et al., 2004; Rushworth et al., 2007; Assadi et al., 2009; Westbrook and Braver, 2016). In summary, a plethora of empirical studies show modulation of ACC activity by the mesolimbic dopaminergic pathway, and this modulation seems to play a crucial role in determining the direction and intensity of effort expenditure.

The third line of evidence shows that one of the outputs of the Salience Network is closely related to the activity of the sympathetic system. The most objective measurements of effort rely on indices of sympathetic outputs, such as pupillary dilatation (van der Wel and van Steenbergen, 2018) or the cardiac pre-ejection period (Richter et al., 2008). We assume that if the Salience Network generates the effort signal necessary to perform a cognitive task or a physical exercise, it should simultaneously send a signal to the sympathetic nervous system to expend the energy necessary to achieve the planned goal (see Figure 2).

In a series of experiments, Hugo Critchley and his colleagues clearly demonstrated the direct involvement of the dorsal ACC in the control of autonomic arousal during volitional behaviors, including effortful cognitive processing (for a review see Critchley, 2005). Using a similar approach, Julian Thayer and his coworkers proposed a neurovisceral integration model that clearly establishes a link among cognitive performance, heart rate variability (HRV) and a network involving the prefrontal cortex, the ACC and the insula (Thayer and Lane, 2000, 2009; Thayer and Friedman, 2002; Thayer et al., 2009). In summary, several lines of evidence establish a clear association between the activity and integrity of the Salience Network and autonomic outputs, such as HRV and pupil size, which are two typical indices of effort investment.

The fourth line of evidence shows that the control signal generated by the Salience Network precedes the activation of other networks involved in task performance such as the CEN. Indeed, if we assume that the effort signal determines the direction and intensity of goal-oriented actions, all brain structures involved in the achievement of these actions during a task should receive this effort signal to participate in their execution. Sridharan et al. (2008) showed that the right anterior insula plays a critical and causal role in switching between the DMN and the CEN. These authors used Granger causality analyses to examine the causal interactions between the Salience Network and other brain networks, such as the DMN and the CEN, by assessing the extent to which the signal changes in one brain region could predict the signal changes in another brain region. These authors showed that the right anterior insula activates the CEN and deactivates the DMN across various task paradigms and stimulus modalities while participants perform a task. Similarly, Menon and Uddin (2010) suggested that the right anterior insula is involved in switching between the DMN and CEN and acts as a “causal outflow hub” coordinating these two major large-scale networks (see Figure 2). The involvement of the Salience Network in the neural process underlying the switch between the DMN and the CEN has been confirmed by two other teams (Wen et al., 2013; Goulden et al., 2014).

In addition, neuroimaging studies using a Granger causal analysis provided evidence that individuals with schizophrenia exhibit a reduction in the strength of the causal influences from the right anterior insula on the CEN and DMN (Moran et al., 2013; Palaniyappan et al., 2013; Manoliu et al., 2014). This pattern of abnormal connectivity among the three main large-scale networks suggests that structural and functional abnormalities in the insula are components of the neuropathology of schizophrenia (Uddin, 2014), which is a disorder well-known to be associated with a lack of motivation and reduced effort (Culbreth et al., 2018). In summary, strong evidence confirms the role of the Salience Network in coordinating and controlling the deactivation of the DMN and the activation of the CEN during task performance.

The fifth line of evidence shows that lesions and dysfunctions in the Salience Network lead to a lack of effort-based decision-making. Three sets of neuropsychological data support this assumption. First, Cohen et al. (1999) reported that patients who underwent bilateral cingulotomy for the treatment of intractable pain showed significant impairment in focused attention, intention, and executive functioning associated with spontaneous response production 12 months after the surgical intervention compared with baseline. Second, fronto-temporal dementia is a neurodegenerative disorder that generally emerges during the sixth decade of life and selectively affects von Economo neurons (VENs) as demonstrated in post-mortem quantitative neuroanatomical studies (Seeley et al., 2006; Kim et al., 2012; Santillo et al., 2013). A loss of more than 50% of VENs was observed in patients with fronto-temporal dementia in the three cited post-mortem studies. Individuals with fronto-temporal dementia exhibit several cognitive and emotional impairments, including a loss of initiative and deficiencies in self-control. Third, apathy has been conceptualized as a motivational impairment or deficit in goal-directed behavior with a decrease in emotional involvement and difficulty in initiating new actions (Duffy, 2000; Levy and Dubois, 2006). According to Le Heron et al. (2018), apathy is strongly associated with disruption in the dorsal ACC and ventral striatum, which includes the nucleus accumbens, in several brain disorders, such as Parkinson’s disease, Alzheimer’s disease, Huntington’s disease, and stroke. These results support the hypothesis that the two main hubs of the Salience Network, i.e., the anterior insula and ACC, play a crucial role in initiating motivated and effort-based goal-directed behaviors.

The empirical data reviewed in the preceding paragraphs clearly support the hypothesis that the Salience Network underpins the mechanism of effort. The second aim of “Neurophysiological Arguments for an Integrative Model of Effortful Control” section is to describe the inherent organization of the Salience Network to more precisely define the resources required to compute and sustain the effort signal over time.

### Modular Organization of the Dorsal ACC and Anterior Insula Cortices

In “Definitions of Key Concepts” section, we proposed that a limited number of processing units dedicated to the mechanism of effort generate the effort signal. In this section, we assume that the cortical minicolumns composing the dorsal ACC and anterior insula are the neuronal substrates of the effort-dedicated processing units.

In all mammalian species, including humans, the neocortex is a cellular sheet composed of pyramidal neurons and interneurons deployed in horizontal layers intersected by vertical columns (Mountcastle, 1995; Defelipe et al., 2012). According to the Pasko Rakic’s radial unit model, proliferative units located in the ventricular and subventricular zones produce all neocortical neurons during the first half of gestation (Rakic, 1988, 2009). Neurons generated in a single proliferative unit form a single morphologically identifiable stack of neurons in the cortex termed the “ontogenetic” column, which becomes a cortical minicolumn during adulthood (Mountcastle, 1997; Buxhoeveden and Casanova, 2002). The number of ontogenetic columns in specific cytoarchitectonic areas can be expected to vary across species and individuals. In humans, the total number of ontogenetic columns throughout the neocortex is estimated to range between 150 and 200 million with a probable high individual variability. Consequently, the adult neocortex can be conceived as a mosaic of interrelated minicolumns or radially organized modules of neurons. By convenience, we called “cortical columns” these minicolumns throughout the manuscript. The number of columns determines the size of the cortical surface whereas the number of neurons in a column determines its thickness.

The cortical columns consist of an array of iterative neuronal groups that extend radially across cellular layers 6–2 with layer 1 at the top. The neurons in a given column are stereotypically interconnected in the vertical dimension, share extrinsic connectivity, and hence act as basic functional units subserving a set of common static and dynamic cortical operations (Rakic, 2008). These operations include not only sensory and motor functions but also the highest cognitive functions, such as executive functions. The radial unit model also postulates that the number of ontogenetic columns devoted to a given area can be further regulated by afferents from subcortical and other cortical areas, particularly during a critical or sensitive period of the brain maturation process. According to the radial unit model, we hypothesize that the dorsal ACC and anterior insula cortical areas comprise a finite and limited number of cortical columns that are highly specialized in effort regulation and that each cortical column is a processing unit subserving the mechanism of effort and participating in the generation and maintenance of the effort signal.

### Intrinsic Organization and Specificity of the Cortical Columns in the Dorsal ACC and Anterior Insula

The mechanism of effort and its related neuronal Salience Network are essential for the regulation of high-level cognitive functions such as executive functions. We assume that this mechanism is at the top of the hierarchical organization of the nervous system. Therefore, the mechanism of effort should possess several structural and/or functional characteristics that distinguish it from other brain systems. Two main singularities are pointed out hereafter.

First, the cortical columns in the dorsal ACC and anterior insula present differences in laminar structure in comparison to most other cortical areas. While most cortical areas have six well-delineated layers (eulaminate cortex), the dorsal ACC and anterior portion of the insula contain no aggregates of granule cells and lack layer 4 (agranular cortex) or have a poorly formed layer 4 (dysgranular cortex; Bonthius et al., 2005; Barbas and García-Cabezas, 2016). Concerning the dorsal ACC, the agranular area corresponds to Broadman’s area 24, whereas the dysgranular area corresponds to Broadman’s area 32 (Palomero-Gallagher et al., 2008). The dorsal ACC is also characterized by a prominent layer 5, which can be subdivided into layer 5a with numerous densely packed large pyramids and a cell sparse layer 5b (Palomero-Gallagher and Zilles, 2019). Agranular and dysgranular areas have a lower density of neurons than eulaminate areas, especially in the upper layers (Barbas and Pandya, 1989; Barbas and García-Cabezas, 2016).

Limbic areas, such as the dorsal ACC and anterior insula, have a competitive advantage over eulaminate areas because they widely connect with a variety of subcortical structures that develop before the cortex (Barbas and García-Cabezas, 2016). Such areas do not receive driving thalamocortical inputs into layer 4 as is the case in eulaminate areas (e.g., primary visual cortex). This absence of specific inputs arriving in layer 4 may facilitate the mixing of various incoming signals from the whole brain (Wylie et al., 2015), such as cost-benefit signals with regard to the dorsal ACC and anterior insula. A recent hypothesis states that limbic areas, including the dorsal ACC and anterior insula, are at the top of the predictive hierarchy in all cortical systems. The main role of limbic areas is to send prediction signals to downstream areas, while most laminated areas (e.g., primary sensory cortices) are at the lowest level of the hierarchy and receive these prediction signals (Chanes and Barrett, 2016). Prediction signals can be viewed as controlling or modulating signals that shape the processing of target brain areas.

Second, the dorsal ACC and anterior insula are further distinguished from eulaminate cortical areas due to the following important feature: these areas contain large bipolar glutamatergic neurons named VENs located in layer 5b in clusters of 3–6 neurons (Allman et al., 2011; Butti et al., 2011; Dijkstra et al., 2018). The vertical orientation of VENs and the narrow lateral extent of their dendritic arbors suggest that these neurons may relay the output of cortical columns (Watson et al., 2006). Several empirical arguments suggest that VENs bear large, rapidly conducting axons (Watson et al., 2006). Altogether, this evidence suggests that the function of VENs may be to provide a rapid relay to other parts of the brain of a simple signal derived from information processed within the anterior insula and dorsal ACC. VENs are present in great apes but absent in other primates (Nimchinsky et al., 1999; Allman et al., 2011). This distribution suggests that VENs contribute to specializations of neural circuits in species that share both a large brain size and complex social cognition (Stimpson et al., 2011). In contrast to pyramidal neurons and fusiform neurons in layer 6, the somatic volume of VENs is strongly correlated to the encephalization quotient in both humans and great apes (Butti et al., 2011), which may reflect a possible link between VENs and some cognitive functions supported by the Salience Network. For instance, VENs are viewed as the neuronal basis of switching processes between the CEN and DMN (Sridharan et al., 2008) or as the motoneurons of the cortico-autonomic pathway (Butti et al., 2011). VENs have been found to be absent or dysmorphic in various disease processes such as autism (Santos et al., 2011), schizophrenia (Brüne et al., 2011), and agenesis of the corpus callosum (Kaufman et al., 2008). These three disorders are related to alterations in effort-based decision-making (Brown et al., 2012; Damiano et al., 2012; Fervaha et al., 2013; Gold et al., 2013; Treadway et al., 2015; Mosner et al., 2017; Culbreth et al., 2018).

In addition to these specificities, the cortical columns in the Salience Network share the following commonality with other cortical columns throughout the brain: they include two main categories of neurons called pyramidal neurons and inhibitory interneurons. Pyramidal neurons are glutamatergic neurons mainly found in layers 2–3 and 5–6 of the agranular and dysgranular columns. The pyramidal neurons in layers 2–3 are involved in intra columnar corticocortical communication (Medalla et al., 2017). These neurons can also send information horizontally within their lamina through long-range tangential connections to excite neighboring columns (Thomson and Bannister, 2003). The simultaneous activation of interconnected pyramidal neurons in layers 2–3 belonging to the same network could contribute to a global workspace supporting awareness (Dehaene et al., 1998; Dehaene and Changeux, 2011). We assume that layer 2–3 pyramidal neurons in cortical areas of the Salience Network participate in the generation of the effort signal, and their synchronous activity within several cortical columns, such as those of the dorsal ACC and anterior insula, could contribute to the feeling of effort. The anterior insula has been clearly associated with interoceptive awareness and subjective feeling states (Critchley et al., 2004), whereas anterior insula and dorsal ACC have been identified respectively as input and output components of a system based on awareness of self (Medford and Critchley, 2010). Other brain regions can also participate in the feelings of effort and fatigue according to the nature of the task (e.g., cognitive vs. physical). For instance, significant positive relationships were found between brain activity in cerebellar, temporal, cingulate and frontal regions and subjective mental fatigue in a challenging working memory task designed to induce mental fatigue (Cook et al., 2007).

The pyramidal neurons in layers 2–3 have a small soma but a high density of spines and dendrites (Sasaki et al., 2015), which certainly integrate all interoceptive inputs from the thalamus and other subcortical regions. Elston et al. (2005) noted that layer 3 pyramidal cells in the ACC are on average at least eight times more spinous than those in the primary visual cortex in the same hemisphere. This characteristic of layer 2–3 pyramidal neurons in the dorsal ACC and anterior insula suggests that these neurons are good candidates for receiving incoming cost-benefit signals from other brain regions.

**Figure 3:**
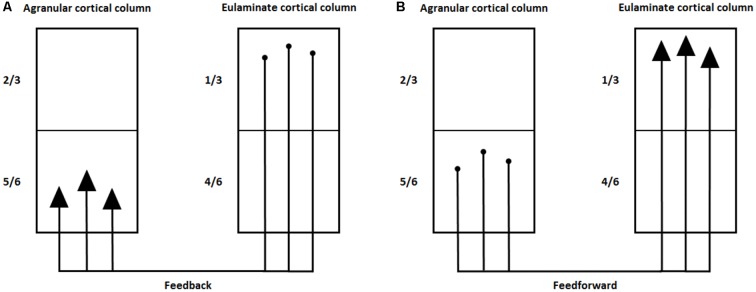
Connections between agranular and eulaminate cortical columns. **(A)** Feedback pathway from layer 5–6 pyramidal neurons in agranular columns to layers 1–3 in eulaminate columns. **(B)** Feedforward pathway from layer 1–3 pyramidal neurons to layers 5–6 in agranular columns.

Upper layer small pyramidal neurons form vertical connections with larger pyramidal neurons in layers 5–6 that generate most of the output from the neocortex to other cortical/subcortical parts of the brain (Opris et al., 2017). The pyramidal neurons in layers 5–6 of agranular and dysgranular cortical columns mainly project to layer 1 of eulaminate cortical columns (feedback pathway). Reciprocally, the pyramidal neurons in layer 3 of eulaminate cortical columns project to layers 5–6 of agranular and dysgranular cortical columns (feedforward pathway). Figure 3 illustrates this bidirectional connectivity between cortical columns. Consequently, we can expect that layer 5–6 pyramidal neurons in the dorsal ACC and anterior insula send the effort signal to other brain regions to control and modulate their activity.

The cortical columns also contain inhibitory neurons that project to the white matter to contact distant brain regions and participate in local circuits in the same cortical column. These neurons generally target distinct morphological compartments of pyramidal neurons, such as the soma, dendrites and axon. Inhibitory interneurons generally use gamma-aminobutyric-acid (GABA) as their main neurotransmitter. Their functions include the regulation of the gain and dynamic range of excitatory pyramidal outputs, the establishment of the time window for the reception of inputs and participation in the brain rhythmic patterns of neural activity (for a review, Buzsáki et al., 2007), particularly during the long periods of silence observed in slow brain frequency as described in the following section.

### The Effort Signal as a Product of the Synchronized and Rhythmic Firing of Pyramidal Neurons

The first purpose of this section is to delineate the neurophysiological mechanism that explains how the mechanism of effort generates and maintains the effort signal over time. The second purpose of this section is to specify the characteristics of the effort signal. Previously, we proposed that the cortical columns of the Salience Network compute and transmit the effort signal to other cortical areas and brain structures to achieve the intended goal. If this signal has to be maintained over time in the absence of an external stimulus by the simple power of the will, the assembly of neurons involved in its generation must have the intrinsic property to maintain a pattern of electrocortical activity during the whole task. Several studies suggest that sustained attention in cognitive as well as motor tasks is associated with a pattern of very slow oscillatory electrocortical activity above cingulate regions (Onton et al., 2005; Sauseng et al., 2007; Kao et al., 2013).

Convergent data demonstrate that slow oscillatory activity is generated by pyramidal neurons of the ACC (e.g., Voloh and Womelsdorf, 2018), certainly layer 2–3 pyramidal neurons. The neurons in layers 2–4 have long-range cortico-cortical tangential connections that allow several cortical columns sharing the same function to synchronize their activity. Layer 2–3 pyramidal neurons preferentially fire synchronously at a low frequency, while layer 4 pyramidal neurons (absent in ACC) preferentially fire at a high frequency (Lachaux et al., 2005). This rhythmic firing causes fluctuations in cortical local field potentials that can be measured using implanted electrodes (e.g., intracranial electroencephalography—EEG) or scalp detectors (e.g., scalp EEG or magnetoencephalography—MEG).

**Figure 4:**
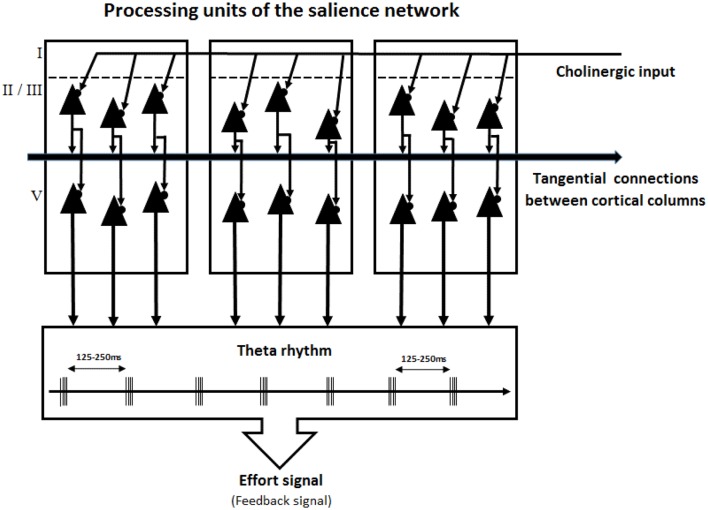
The Salience Network as a slow-wave generator. The processing units in the Salience Network are the cortical columns of the dorsal ACC and the anterior insula. Layer 2–3 pyramidal neurons of these cortical columns generate a theta rhythm (4–8 Hz) under the control of cholinergic input. Tangential connections between cortical columns allow processing units to pulse synchronously. Layer 5 pyramidal neurons transmit this slow-wave inhibitory control signal to eulaminate brain regions to focus their processing on relevant task features.

Brain oscillation frequencies are divided into the following spectral bands with distinct functional associations: delta (1–4 Hz), theta (4–8 Hz), alpha (8–14 Hz), beta (14–30 Hz), and gamma (>30 Hz). Lakatos et al. (2005) proposed that these oscillations are hierarchically organized as follows: cortical columns that generate low-frequency oscillations (theta and alpha bands) modulate the activity of cortical columns that generate higher-frequency oscillations (beta and gamma bands). Gamma band oscillations are short bursts of high-frequency action potentials separated by short periods of 10–30 ms, whereas theta-band oscillations are short bursts of high frequency separated by long silence periods of 125–250 ms (Figure 4). The periods of silence can be viewed as inhibition periods during which layer 2–3 pyramidal neurons do not generate any action potentials.

In fact, neural populations and cortical columns can oscillate in phase (i.e., synchronously) or out of phase (i.e., asynchronously) with one another. The network connectivity defined in “Definitions of Key Concepts” section is higher when two networks oscillate synchronously and weaker when they oscillate asynchronously. According to the Hebb principle (Hebb, 1949), if several synapses participate in the oscillations of the same network, they mutually reinforce each other. When oscillation is out of phase, the cortical columns do not communicate optimally because the action potentials from one column arrive when the activity of the other is inhibited (for reviews, see von Stein and Sarnthein, 2000; Clayton et al., 2015). This principle of synchronized firing between several brain regions has been termed “communication through coherence” (Fries, 2005, 2015). According to this principle, interregional communication is established when the oscillatory activity in two ensembles of cortical columns is coherent, i.e., they oscillate at the same frequency with a stable phase difference.

Furthermore, several studies have shown that communication between networks is optimal when the activity of cortical columns firing in gamma band is synchronized with the activity of cortical columns firing at low frequencies (Canolty et al., 2010; Oehrn et al., 2014; Hoy et al., 2016; Siebenhühner et al., 2016). This second principle of the synchronization of a high-frequency brain region and a lower frequency brain region has been termed “gating by inhibition” (Jensen and Mazaheri, 2010). According to this principle, the information flow between brain regions is established by actively inhibiting the pathway not required for the task (Bonnefond et al., 2017). Based on this perspective, alpha and theta band activity could reflect pulses of inhibition, and the brain regions that generate these two rhythms could be a part of inhibitory control systems. Notably, the ACC has been clearly identified as a theta rhythm generator (Leung and Borst, 1987; Wang et al., 2005; Voloh and Womelsdorf, 2018). In addition, Voloh et al. (2015) showed that failures in attention shifting are associated with decoherence of theta to gamma interactions in a network comprising the ACC and the lateral prefrontal cortex, while the former exerted control over the latter. We assume here that the theta rhythm is the main signature of the effort signal generated by the Salience Network that exerts control over other task-related brain areas. Theta oscillations would play the role of filtering information in the brain regions receiving the effort signal. Theta oscillations would facilitate and prolonge the action of the pertinent signals entering the concerned brain region in phase with concomitantly short bursts of high frequency (filtering in) and prevent the processing of non-pertinent signals appearing during the periods of silence (filtering out).

The theta rhythm has been associated with mental fatigue (Boksem et al., 2005; Borghini et al., 2014; Wascher et al., 2014). A decrease in the theta band generated by the Salience Network could be associated with a decrease in inhibitory control over downstream brain regions, and an increase in the theta band could be an index of engagement in effortful control. Neuropsychological studies confirm this hypothesis. For instance, decreases in task-related theta band activity have been regularly reported in schizophrenic patients (Popov et al., 2015; Roa Romero et al., 2016; for a review, Galderisi et al., 2009) who have long been associated with motivational impairment and aberrant effort-based decision-making (Culbreth et al., 2018). More interestingly for the purpose of this section, theta activity is observed throughout the entire duration of a vigilance task in humans (Boksem et al., 2005; Wascher et al., 2014). Therefore, we assume that variations in effortful control during an experimental session can be indexed by theta rhythm EEG recorded above the mid frontal brain area.

The theta rhythm has also been associated with conflict detection and monitoring (e.g., Töllner et al., 2017). Furthermore, several studies showed that the dACC is activated in case of response conflict (e.g., Kim et al., 2012). The conflict refers to situations that require overriding prepotent responses, selecting among a set of equally permissible responses or making errors. Response conflict is often associated with top-down and effortful control (Botvinick et al., 2001). Frontal theta oscillation is generally envisaged as a mechanism for cognitive control (Cavanagh and Franck, 2014) and more specifically effortful control (Vassena et al., 2017), rather than a response to increased conflict. In this regard, there is an extensive literature showing that frontal midline theta oscillations correlate positively especially for tasks that demand sustained control (for review, see Holroyd and Umemoto, 2016) even in the absence of response conflict (Mulert et al., 2005; Aarts et al., 2008; Vassena et al., 2014).

In accordance with this evidence, we assume that during a cognitive task or an exercise requiring sustained effortful control, layer 2–3 pyramidal neurons in the dorsal ACC and anterior insula fire in a theta spectral band (4–8 Hz; Figure 4). The apical dendritic trees of layer 2–3 pyramidal neurons extend into layer 1, where they receive three types of inputs that ensure this continuous slow rhythm of oscillations. First, these dendritic trees receive extracolumnar excitatory inputs that participate in recurrent excitatory loops. These loops allow the persistent activity to be sustained in the absence of external inputs within each cortical column. Second, the neurons receive extracolumnar inhibitory cholinergic inputs from basal forebrain nuclei (Geula and Mesulam, 1989; Lewis, 1991; Selden et al., 1998; Ballinger et al., 2016). These cholinergic projections from the basal forebrain to the pyramidal neurons in the dorsal ACC and anterior insula are an important part of the model of attentional effort conceived by Sarter et al. (2006). Third, these neurons receive intracolumnar inhibitory inputs from the ascending axons of Gabaergic Martinotti interneurons (Hof et al., 1999; Tremblay et al., 2016).

The integration of these numerous and miscellaneous inputs by the pyramidal neurons in layers 2–3 produces a sustained theta rhythm (Oddie and Bland, 1998). This theta rhythm spreads through neighboring cortical columns *via* long-range axons that expend horizontally within their lamina. Thus, all cortical columns involved in effortful control can pulse synchronously. Simultaneously, layer 2–3 pyramidal neurons send their pattern of oscillatory activity to layer 5 pyramidal neurons. Using this integrated information, the pyramidal neurons in layer 5 transmit the theta band oscillatory rhythms to downstream executive and/or motor centers. Thus, these neurons can impose a pattern of rhythmic epochs of inhibition to other cortical and subcortical areas.

In the current section, we presented arguments supporting the existence of a rhythmic control signal generated by the Salience Network supporting what we called the effort signal. Based on this background, we next examine several neurobiological mechanisms that can explain why effort can decrease over time due to a weakening of the efficiency of processing units that ensure its deployment.

### Neurobiological Mechanisms Underlying Acute Mental Fatigue

A central point of the integrative model of effortful control is the assumption that the capacity of the effort-dedicated processing units that generate the effort signal can be weakened in the case of prolonged heavy solicitation. The aim of the current section is to present a post-synaptic mechanism at the level of the dendrites of layer 2–3 pyramidal cells in the cortical columns of the Salience Network as an alternative explanation of mental and central fatigue.

According to our approach, the decrease in the capacity of the cortical columns in the Salience Network to generate and maintain the effort signal depends on a progressive alteration in an intracellular signaling mechanism that regulates the electrophysiological properties of the membrane of each layer 2–3 pyramidal neuron belonging to these cortical columns. In the case of a long effortful task or exercise, these pyramidal neurons become less able to sustain the theta rhythm, which is essential as the frame of the effort/control signal, over time. This mechanism can be viewed as a form of short-term neuroplasticity.

*Amy Arnsten* and her team clearly described this mechanism and demonstrated its crucial role in the impairment of high-level cognition such as working memory, when an organism has to cope with uncontrollable stress (for reviews, see Arnsten, 2009; Arnsten et al., 2010, 2012). We assume that similar mechanisms are involved in the case of fatigue induced by an effortful task or an exhausting physical exercise. As previously proposed, the mechanism of effort relies on networks of interconnected pyramidal cells. Arnsten et al. (2010) showed that critical molecular events occurring near each glutamatergic synapse of pyramidal neurons determine the capacity of a network to generate synchronized rhythmic oscillations. The molecular mechanisms identified by Arnsten et al. (2010) occur in the neighborhood of the pyramidal cells in layers 2–3 that receive extracolumnar excitatory glutamatergic inputs from the mediodorsal thalamus. The two subsequent paragraphs describe these transient deleterious mechanisms.

Arnsten et al. (2010) referred to “dynamic network connectivity” (DNC) as the molecular mechanisms that rapidly and transiently modify the electrophysiological properties of the membrane of prefrontal pyramidal neurons through the hyperpolarization-activated cyclic nucleotide-gated (HCN) cation channel current, cyclic adenosine monophosphate (cAMP) modulation and other similar mechanisms. These molecular mechanisms all finely tune the rhythmical oscillations of cortical columns and other subcortical structures. Any serious upregulation or downregulation of these molecular mechanisms induced by a stressful or fatiguing task may lead to a dysregulation of the effort signal or other high-level cognitive functions (i.e., executive functions). For instance, Wang et al. (2007) demonstrated that increased cAMP, in prefrontal pyramidal cells markedly reduces network activity during a delay period and impairs working memory performance.

In the following paragraphs, we focus on three endogenous molecules that dysregulate the rate of production of cAMP within the cytoplasm of pyramidal neurons and deteriorate the capacity of prefrontal cortical columns (i.e., processing units) to synchronously generate an oscillatory control signal. We assume that any serious dysregulation of the intracellular concentration of cAMP could lead to weaker connectivity in the Salience Network and a deterioration of the effort/control signal. Thus, mental/central fatigue could lead to a notable dysregulation of cAMP through different pathways, followed by a detrimental effect on Salience Network connectivity *via* HCN channel activation. By contrast, in normal situations (i.e., no overloading of processing units), the regulation of cAMP is finely and optimally tuned by a moderate concentration of noradrenaline binding alpha2-A receptors (Arnsten et al., 2010).

First, dopamine and noradrenaline levels that are too high generally lead to a decrement in performance in tasks involving the prefrontal cortex, representing the well-known “inverted-U” effect (for a review, Cools and D’Esposito, 2011). Based on this perspective, Arnsten et al. (2010) argued that dopamine D1 receptor stimulation and stressful events weaken prefrontal function by upregulating cAMP and opening HCN channels (Vijayraghavan et al., 2007; Gamo et al., 2015). Similar results are expected with adrenaline, which must bind beta1 receptors in the case of high levels of stress (Ramos and Arnsten, 2007). These two pathways could participate in the fatigue phenomenon in the case of a very stressful task or vigorous exercise. Several studies have shown a high level of intracerebral catecholamines during exercise in animals (Meeusen and De Meirleir, 1995; Pagliari and Peyrin, 1995).

Second, it has been shown in different species and brain regions that serotonin increases the activity of HCN channels through G protein-coupled receptors, which activate adenylate cyclase and *via* a cascade induce the production of cAMP (Bobker and Williams, 1989; Garratt et al., 1993; Ko et al., 2016). In fact, neurons in the raphe nuclei release serotonin into layers 2–3 of the cortical columns in the ACC and insula (see Figure 2). These serotonin molecules may bind 5-HT1 receptors located on the membrane of pyramidal neuron spines. Notably, serotonin has been associated with central fatigue in exhausting exercise, particularly in warm environments (for reviews, see Newsholme et al., 1992; Meeusen et al., 2006).

Third, the dysregulation of the Salience Network may be related to a serious use of glucose or oxygen through the production of adenosine in the case of strong violations of homeostasis. Minor and Hunter (2002) proposed that excitatory transmission is regulated by a “circuit-breaker” mechanism that is directly linked to an imbalance in the energy supply/demand ratio. Such an imbalance could arise from the inadequate delivery of glucose (hypoglycemia) and oxygen (hypoxia) or during excessive excitatory transmission (Meghji, 1991; Fredholm et al., 2005). Either condition results in the rapid hydrolysis of adenosine triphosphate (ATP) into adenosine. The nucleoside is released into the extracellular space and binds specific receptors located on the pre-and postsynaptic membranes of neurons. Adenosine acts as an inhibitory purinergic neuromodulator *via* the activation of G protein-coupled receptors (A1, A2A, A2B, and A3). Adenosine modulates the activity of HCN channels in the nervous system and exerts a profound negative influence on neuronal excitability (He et al., 2014). Therefore, adenosine potently inhibits neural excitation and transmitter release, thereby facilitating the recovery of energy homeostasis (Hoehn and White, 1990; Milusheva et al., 1990; Fredholm et al., 2005). Caffeine is a high-affinity adenosine receptor antagonist that derives its stimulant properties under conditions of fatigue by disinhibiting brain neurons under adenosine regulation (McLellan et al., 2016).

The degree to which these neurotransmitters and metabolites participate in mental/central fatigue is unclear. We can expect that their respective contributions vary according to the type of task (cognitive task, psychosocial task, or physical exercise), its modality of completion (continuous or interrupted by breaks), its intensity/difficulty and its duration (time on task).

The connectivity of the Salience Network can also be modified durably through long-term synaptic plasticity mechanisms. However, these positive and negative structural changes in the connectivity of the Salience Network are beyond the scope of the present article.

In this section, we provided neurophysiological evidence regarding the short-term postsynaptic mechanisms supporting a possible transitory weakening of the efficiency of effort-dedicated processing units with an increase of time on task and mental workload.

## Challenging the Integrative Model of Effortful Control

The rationale delineated by the integrative model of effortful control integrates knowledge from the fields of social psychology, cognitive psychology and neuroscience. The interest of all integrative interdisciplinary models is to formulate new hypotheses that can be tested at different observation levels, such as the phenomenological level, the behavioral level, the psychophysiological level, and the neurophysiological level in the present case. The phenomenological level is related to the measurement of the awareness of effort, fatigue, costs and benefits during and following effortful tasks or exercises through subjective scales and questionnaires. The behavioral level relies on the measurement of performance and behavioral strategies through task-related indices. The psychophysiological level is related to the measurement of indices recorded during effortful tasks or exercises, such as pupillary dilatation and the pre-ejection period. The neurophysiological level corresponds to the measurement of brain-related connectivity or activity during effortful tasks and exercises in humans using brain-imaging techniques such as near-infrared spectroscopy (NIRS), MRI, PET, EEG or MEG. The neurophysiological level also includes animal studies involving single-unit recordings measuring the electrophysiological responses of single neurons and microdialysis allowing for the analysis of the concentrations of endogenous molecules (e.g., neurotransmitters, glucose, adenosine, etc.) in the cerebrospinal fluid. All or some methodological approaches can be combined in the same experiment through interdisciplinary protocols to test hypotheses regarding effortful control.

In the first part of this fourth section, we focus on the measurement of two aspects of effortful control: the effort signal and the feeling of effort. Finally, we examine how it is possible to test the main hypotheses of the present model: acute fatigability of the processing units of the mechanism of effort due to a decrease in connectivity between the Salience network and the CEN. In each part, we propose several avenues of future research.

### Measuring the Effort Signal

Measuring the effort signal could be the best way to infer the computations carried out by the mechanism of effort according to the manipulation of cost-benefit signals. The effort signal can be observed solely at the neurophysiological level. The easiest way to observe fluctuations in the effort signal is certainly the use of EEG to observe spectral variation (i.e., power of theta and alpha rhythm) in the mid-central region of the scalp, just above the ACC, during the first and the second tasks of the sequential protocol over time. This technique has already been used by different authors (Fairclough and Ewing, 2017; Puma et al., 2018) and seems relatively reliable. Another approach is to use the event-related potential (ERP) technique to observe variations in the amplitude of some theta-related ERP components in the second task of a sequential protocol. For instance, Cavanagh and Franck (2014) showed that ERP components such as N2 elicited by novelty or a stimulus-response conflict, feedback-related negativity, correct-related negativity, and error-related negativity, reflect dorsal ACC-related control processes and share a common spectral signature in the theta band. A third approach is to use the single-cell recording in dorsal ACC and anterior insula neurons in animals (e.g., monkeys) during effortful tasks. Several teams in France and Japan have already performed this type of recording in the ACC (Shima and Tanji, 1998; Procyk et al., 2000; Matsumoto et al., 2003).

### Distinguishing the Feeling of Effort and the Feeling of Fatigue

An interesting approach could be to distinguish the feeling of effort from the feeling of fatigue. For this purpose, it could be appropriate to measure several subjective and objective indices of effort during an effortful task in a large population of participants and then conduct a factorial analysis to test the hypothesis that different dependent variables could load on two distinct factors. For instance, subjective scales measuring effort, the pre-ejection period and pupil dilation could load on a first factor named “effort”, whereas subjective scales measuring mental fatigue, and connectivity between the Salience Network and the CEN with fMRI could load on a second factor named “mental fatigue”. In this study, it could be also very interesting to examine on which factor would load the theta rhythm power density recorded above frontal EEG electrodes.

### Demonstrating the Acute Fatigability of Processing Units

At the behavioral level, it is urgent to precisely define the conditions of the occurrence of the acute mental fatigue effect in several categories of tasks (i.e., psychosocial, cognitive and physical tasks). This issue can be addressed by manipulating the complexity/intensity and duration of a first task/exercise while maintaining all other conditions equal in a sequential protocol. Several psychophysiological indices, such as the pre-ejection period and theta rhythm, should be measured throughout the two consecutive tasks to obtain continuous objective indices of effort deployment.

At the neurophysiological level, several types of experiments could be very helpful for testing the hypothesis of the weakening of the capacity to generate the effort signal. First, a protocol using brain MRI with a repeated-measure design (i.e., participants carry out the depleting task and the control task on two different days) could measure the between-network connectivity of the Salience Network at rest immediately after the completion of the second task in a sequential protocol. This experiment could be conducted once behavioral and psychophysiological indices of acute mental fatigue would have been validated.

Furthermore, once the conditions of occurrence of the acute mental fatigue effect well defined, it could be very interesting for basic and applied research, to examine the time course of the recovery curve after periods of rest and the factors that shorten or enhance this recovery.

## Conclusion

The mechanisms underlying the decision to stop or invest less effort in effortful tasks are still under debate. Three main limiting factors of performance in effortful tasks have been separately identified in the literature. The first limiting factor, which has been extensively studied and discussed in the resource-based model of self-control (Baumeister and Vohs, 2016), is related to the amount of energy (e.g., glucose) necessary to expend to achieve the goal of a task (i.e., energetic costs). The second limiting factor, which emerges from alternative approaches of the resource-based model (Kurzban et al., 2013; Inzlicht et al., 2014), is related to a motivational shift toward less costly and more pleasant tasks (i.e., opportunity costs). The third limiting factor, which has been conceived by scholars in the cognitive sciences (Hockey, 1997; Shenhav et al., 2017), is related to the structural constraints of the information processing system, such as the presence of a bottleneck or a limited number of processing units devoted to effortful control (i.e., computational costs). Our model introduces the following fourth limiting factor, which has not been considered in previous approaches: the progressive weakening of effort-dedicated processing units with time on task and overloading. Instead of opposing these different limiting factors and viewing their contribution to performance as exclusive, our model attempts to reconcile these different approaches and assumes that each limiting factor plays a role in effort deployment according to the characteristics of the task. Based on this perspective, energetic costs are more relevant for physical exercises than mental tasks. By contrast, computational costs and particularly the weakening of the effort-dedicated processing units are more prominent in very long and intense (i.e., deep concentration; vigorous exercise intensity) tasks. Finally, opportunity costs are more salient when one or more low-cost and attractive alternative tasks are immediately available. Based on this perspective, the ego depletion effect can be explained as a consequence of the synergistic deleterious effect of several costs, and each cost is weighted by the characteristics of the task.

Future studies should determine the relevance of the different costs according to the task performed by the individual. We are convinced that this new approach could enhance our understanding of failures in effort-based decision-making in everyday life, lapses and relapses of behavior change in health domains, and poor performance in sports achievements.

## Author Contributions

NA, MA and RB conceived the integrative model of effortful control. NA and MA wrote the article. RB supervised the plan of the article.

## Conflict of Interest

The authors declare that the research was conducted in the absence of any commercial or financial relationships that could be construed as a potential conflict of interest.
